# Impacts of health expenditures and environmental degradation on health status—Disability-adjusted life years and infant mortality

**DOI:** 10.3389/fpubh.2023.1118501

**Published:** 2023-03-28

**Authors:** Anis Omri, Bassem Kahouli, Montassar Kahia

**Affiliations:** ^1^Department of Business Administration, College of Business and Economics, Qassim University, Buraidah, Saudi Arabia; ^2^Community College, University of Ha’il, Ha’il, Saudi Arabia; ^3^Department of Finance and Economics, College of Business and Economics, Qassim University, Buraidah, Saudi Arabia

**Keywords:** health status, mortality, environmental sustainability, carbon dioxide, sustainability

## Abstract

**Introduction:**

Human health and well-being are intimately related to environmental quality. In this respect, the present study contributes to the existing health economic literature by examining whether public and private health expenditures (PPHE) moderate the incidences of environmental degradation on the health status in Saudi Arabia, particularly disability-adjusted life years (DALYs) and infant mortality.

**Methods:**

Using the fully modified ordinary least squares (FMOLS) method.

**Results and Discussion:**

The empirical results revealed that (i) unconditional positive impacts of CO_2_ emissions on increasing DALYs and infant mortality; (ii) conditional negative impacts of public health expenditures on DALYs and infant mortality in all the estimated models, whereas global and private expenditure contribute only on reducing infant mortality; (iii) public health expenditure is more effective than private health expenditure in reducing infant mortality; (iv) the effects of the interactions between the indicators of both health expenditures and CO_2_ emissions on DALYs and infant mortality are negative and significant only for the specifications relating to public health expenditures, indicating that this later could be employed as a policy or conditional variable that moderates the adverse impacts of carbon emissions on the population’s health status. Generally, the study presents an overview of environmental health change’s effects and examine how these effects may be reduced through increasing health spending. The study provides recommendations for addressing health status, health expenditures, and carbon emissions, all of which are directly or indirectly linked to the study.

## 1. Introduction

As a fundamental universal right, health is moreover a major resource for social and economic progress. The World Health Organization (WHO) revealed that all peoples of the world have the right to the greatest attainable typical of health. Health is not only the nonexistence of malady or disability, but also mental, physical, and social welfare. The health sector is the first basic social sector for all countries. An unhealthy environment poses health risks and then a violation of the right to health. In this context, Gwangndi et al. (1) argue that environmental quality directly influences human well-being and health status, whether in urban cities or in the hinterland and it has been established that environmental degradation due to human activities could lead to malnutrition and mortality, morbidity, and shortened life expectancy. It not only hinders health status but also increases health expenditures (2). The present work extends this debate by investigating the effectiveness of health expenditures in modulating the incidences of carbon emissions on the population’s health status in Saudi Arabia.

Saudi Arabia pays particular attention to the health sector to enhance the health situations of citizens and residents in general and to protect disabled persons in particular. This is in line with the country’s 2030 vision, aimed at guaranteeing a healthy life, supporting the well-being of everyone at all ages and making cities, and ensuring that cities and human settlements are inclusive, secure, durable, and sustainable. However, the rapid urbanization, industrial, commercial, and agricultural evolution of the country has worsened the environment (3). In terms of CO2 emissions, the country is ranked among the top worldwide emitters since its economic activities still depend on traditional energy use and production, causing therefore negative impacts on health status. Moreover, the rapid expansion of oil refineries, land transport, manufacturing companies, and use of pesticides, among others, could increase food, water, and air pollution, which may be, in turn, the invisible cause of certain serious illnesses, such as cancer, birth defects, and other potential hazards to public health (4).

In light of the above motivations and arguments, our study offers several theoretical and empirical contributions to the existing studies. First, from a theoretical viewpoint, health expenditures ensure that public health systems reduce disease risk through a healthy natural environment and promote the green productivity of the economy. They can therefore be an essential factor influencing the quality of the environment. It is therefore substantial to assess the health costs of environmental degradation for the development of both health and environmental policies. Accordingly, the present study seeks to examine the effectiveness of health expenditures in enhancing the population’s health status by mitigating environmental degradation. Second, from a methodological viewpoint, despite the growing interest in the environment-health nexus, there is still much to consider in the relationship between environmental degradation and healthcare status, as worldwide anthropogenic emissions due to consumption and production increase in magnitude (5). Unlike prior studies, this inquiry examines for the first time the moderating effect of health expenditures on the environmental degradation-health status (disability-adjusted life years and infant mortality) nexus. Health expenditure has been considered a policy variable that modulates the negative effects of environmental degradation on health status. This later delivers efficient estimates in small sample sizes and makes it possible to deal with the endogeneity problem of the regressors and the problem of autocorrelation. Both aggregated and disaggregated levels of health expenditures are used to examine their influences on reducing environmental degradation for improving health outcomes. To the authors’ knowledge, this work is the first to handle such research in this context. It is also the first that analyzes the role of health expenditure in improving environmental quality within the Saudi economy. It, therefore, provides new knowledge to support the establishment of health and green policies in line with the natural environment. Third, in terms of policy viewpoint, our results could be specifically helpful for the Saudi decision-making charging for environmental and healthcare issues and provide some degree of imperative comprehension to other countries with health expenditures and development levels comparable to the Saudi economy.

The rest of the study will go as such. Section two presents the literature review on the relationship among health expenditure, environmental degradation, and healthcare status. Section three explains the data, the econometric model specifications, and the procedures we use to estimate the specified goals. Section four presents and discusses the significant findings and their relevance for policy formulation. Finally, section five contains the study findings and offers some policy implications.

## 2. Literature review

A large number of papers have recently been interested in exploring the incidences of environmental degradation in human healthcare [e.g., (4, 6–10)]. For instance, the WHO argue that CO2 emissions are the leading causes of environmental pollution and climate change that negatively affect the population’s health status in different ways, such as through inhalation, skin contact, and ingestion *via* eye contact. WHO also indicates that over the 2030–2050 period, climate change, caused by increased carbon emissions, is expected to lead to around 250,000 additional deaths per year, according to the same source. In this direction, Zeng et al. (11) examined the contributions of socioeconomic situations, physical environment, and air pollution on the health and survival of seniors in China and they showed that air pollution and low seasonal temperatures augmented the risk of disability, mortality, and health deficits. In the same direction, Owusu and Sarkodie (12) studied the influence of air pollution on disability-adjusted life years (DALYs), mortality, and welfare and they found significant positive and negative impacts of environmental pollution on health status (DALYs, premature deaths, and mortality) and economic development, respectively. Moreover, some recent research has examined the role of health expenditures in developing the population’s health status and curbing environmental degradation [e.g., (4, 10, 13–18)]. For instance, Houeninvo (16) examined the consequences of private and public health expenditures on health status for 37 African countries and his results show that public health expenditure effectively improves health status. On the other hand, Ganda (15) examined the role of health expenditure in reducing CO2 emissions for the BRICS countries and he show that both aggregate health expenditures and private health expenditures reduce CO2 emissions, suggesting the need to redesign health spending sub-policies programs to achieve zero carbon goals. The following are the main gaps shared by earlier studies. The first one is linked to the type of relationship among health expenditures, environmental degradation, and the population’s health status. Most prior works have examined either the incidences of CO2 emissions on health outcomes [e.g., (4, 9, 10, 19)], effects of health expenditure on health status [e.g., (13, 14, 16–18)], or the nexus between health expenditure and environmental quality [e.g., (15, 20–22)]. However, to the knowledge of the authors, no earlier study has considered these concepts in one study. The second one is the common failure to consider a policy variable that moderates the consequences of CO2 emissions on health status. In this context, we present below the main works dealing with the relationship between environmental degradation and health. In addition, we also review the major works that deal with both the relationship between health expenditures and environmental degradation as well as between health expenditures and health status.

### 2.1. Environmental degradation and health

There is an abundant literature on the linkage among environmental degradation and healthcare status. It is not possible to make an exhaustive review here, which is why we will present very briefly the most relevant to our research. Starting with multilevel logistic models applied in China, Zeng et al. (11) analyzed the effects of socioeconomic situations, physical environment, and air pollution on health. They found that air pollution and low seasonal temperatures augmented the risk of disability, mortality, and health deficits. By applying the Vector Error Correction Model (VECM) technique, Sinha (23) examined the causal links among industrialization, environmental pollution, and infant mortality rate (IMR) for India over the period 1971–2010 and their findings revealed bidirectional causal links IMR and environmental pollution. In the case of 12 Southern African Development Community (SADC), Mutizwa and Makochekanwa (24) investigated the influence of environmental degradation (CO_2_) on IMR with data spanning from 2000 to 2008 by employing static panel estimators (fixed and random effects). The empirical results show that environmental indicators contribute to 38% of mortality. In the same direction, for 66 low-income countries, Chuang et al. (25) explored the interlink between ecological footprints (EFP), environment degradation, IMR, and U5MR during the period 1980–2010 by using linear mixed structures. They found that EFP and environmental degradation does not have any influence on the link among economic features and health status. Furthermore, Fotourehchi (26) examined the impacts of particulate matter 10 (PM 10) and CO_2_ emissions on IMR for 60 developing countries for the period 1990–2010 by employing a simultaneous equations model. The findings revealed that the improvements achieved in health outcomes by enhancement of socio-economic situations could be lost by PM-10 and CO_2_ emissions. By employing the same method for 30 Chinese provinces, Lu et al. (27) examined the association between carbon emissions, GDP, and public health expenditures during the 2002–2014 period. The empirical results revealed an inverse impact of CO_2_ emissions on public health. In an interesting study, Majeed and Ozturk (8) explored the link among CO_2_ emissions and IMR in 180 nations from 1990 to 2016 by employing several estimation techniques, such as fixed effects, two-stage least squares, and system-GMM estimators. Their findings revealed that CO_2_ emissions generate high IMR. In the same vein, Maiti and Jadhav (28) studied the relationship between three outside pollutants (pollutant minimization program, ozone, and hazardous air pollutants), mortality rate, and DALYs in 164 countries. Empirical findings revealed that the impacts of pollution, deaths, and DALYs rate caused by these outside pollutants are not uniform. Using data for the Middle East and North African (MENA) region, Bouchoucha (29) explored the link among environmental degradation and human health by applying FMOLS and DOLS technologies. The findings show that CO_2_ emissions affect inversely health outcomes. More recently, the study of Arafat et al. (30) analyzed the causality relationship between CO_2_ emissions, life expectancy, and IMR in the case of Pakistan by applying causality cointegration and causality methods. The causality analysis findings showed the occurrence of a unidirectional link moving from CO_2_ emissions to life expectancy and IMR. Similarly, Omri et al. (4) investigated the contribution of research and development in clarifying the link between environmental quality and health status for Saudi Arabia with data spanning from 2000 to 2018 and their results confirmed the negative effect of carbon emissions on health outcomes.

### 2.2. Role of health expenditures

#### 2.2.1. Health expenditures and environmental degradation

Certainly, the extent of environmental degradation will influence the population’s health status, which, in turn, increases health expenditures (4). Several research studies have explored this topic by employing various econometric tools. Most of them have attained the same finding that increasing health expenditures will reduce environmental degradation, particularly CO_2_ emissions. For instance, by employing wavelet analysis for Taiwan, Wu et al. (31) investigated the relationship among environmental quality and health expenditures during the 1995Q1-2016Q4 period and they found a causality linkage among the two variables. Before 2004, findings revealed positive causality running from health spending to environmental degradation. However, before 2007, results revealed negative causality moving from health spending to environmental degradation (long-term). For the BRICS economies, Ganda (15) explored the impact of healthcare spending on CO_2_ emissions for the period 2000–2017 by using FMOLS, VECM, and Dumitrescu-Hurlin causality techniques. Regarding total and private health spending, the empirical results revealed a negative association with environmental degradation. Nevertheless, public healthcare spending is positively correlated with CO_2_ emissions. Besides, causality test results approved bidirectional causality between most of the categorical variables of health spending levels (total, private, and public) and CO_2_ emissions. For seven emerging countries, Bu and Ali (22) investigated the effects of health expenditure, GDP, population, and education on CO_2_ emissions and they found that health expenditure declines the levels of CO_2_ emissions and subsequently improve environmental quality. In the same direction, using the Fourier ARDL (Autoregressive Distributed Lag) model, Li et al. (32) examined the relationships among healthcare spending, environment degradation, and economic growth for the BRICS economies and they found a negative association among environmental degradation and healthcare spending only for India.

#### 2.2.2. Health expenditures and health status

Previous research has made efforts to evaluate and develop the key factors affecting health status and its connection with them. In this subsection, we will review previous studies on the link among healthcare expenditures and populations’ health status. Many scholars found that the augmented health expenditures improve health status, such as life expectancy, under-five mortalities (U5MR), infant mortality rate (IMR), disability, and overall death rate [e.g., (13, 14, 16–18, 33)]. However, some other scholars found confusing or unimportant associations among health expenditures and health status [e.g., (34–36)]. For example, Novignon et al. (33) conducted research for the Sub-Saharan Africa (SSA) countries to examine the impacts of private and public health expenditures (PPHE) on health status between 1995 and 2010. Their findings show that PPHE strongly positively affects health status finished by enhancing life expectancy and reducing IMR. In a similar vein, Karyani et al. (37) compared the influence of PPHE on IMR for Eastern Mediterranean Region (EMR) between 1995 and 2010 by using random effects estimators and they found that public health expenditures reduce IMR; however, private health expenditures do not affect IMR. Furthermore, Nicholas et al. (34) inspected the contribution of PPHE on health outcomes [IMRR, U5MR, and maternal mortality (MMR)] for 40 SSA economies from 2000 to 2010. The empirical findings indicated that public health spending affects negatively and strongly IMR and U5MR, respectively; however, its effect on MMR is negative and insignificant. Raeesi et al. (18) also explored the link among PPHE and three health outcomes indicators (IMR, U5MR, and life expectancy) for 25 countries from 2000 to 2015 and they found a strong link has been observed among PPHE and health outcomes. The contribution of private health expenditure on health status is much higher than public health expenditure. In the same vein, Rezapour et al. (35) studied the impacts of PPHE on IMR, U5MR, and life expectancy as health indicators for selected countries from 2000 to 2015 and their findings revealed that public health spending declines IMR and U5MR, on the one hand; and improve life expectancy, on the other hand. As for private health spending, it has an insignificant effect on the health indicators. For Vietnam, Nguyen et al. (38) explored the associations among disability, health service, and health expenditures. The empirical results indicated that disabled persons had several features of susceptibility (older, less chance of being working, inferior instruction, and poorer) than a person without disabilities. These features have linked with inferior health and advanced need for healthcare use. However, after directing these aspects, disability still had an independent link with advanced health. In the same direction, the main objective of Danovi et al. (39) is to explore the link between lost years due to disability, life expectancy, and health spending in the United States, EU, and several emerging countries in 2017. The findings show that health expenditures have an exponential pace concerning the total of lost years due to disability. Recently, Houeninvo (16) examined the consequence of PPHE on health outcomes (IMR and child mortality) for 37 African countries from 1995 to 2018. System-GMM estimator results show that public health expenditures effectively contribute to reducing mortality. Moreover, Singh et al. (36) investigated the dynamic relationship between PPHE and health outcomes in Southeast Asia by applying the fixed effect, random effect, feasible generalized least squares, and seemingly unrelated regression techniques. Empirical results specified that only public health spending participates in enhancing life expectancy, reducing U5MR levels and mortality rates from non-communicable diseases.

In light of the above-discussed studies, it is clear that most of them have examined either the impact of environmental quality on health status, the impact of health expenditure on health status, or the nexus between health expenditure and environmental degradation. However, slight consideration has been given to the three concepts in one framework. In other words, none of them have examined the effectiveness of health expenditures in reducing the effects of CO_2_ emissions on health status (infant mortality and disability-adjusted life years).

## 3. Methodology and data

### 3.1. Model specifications

The concern to enhance the population’s health status occupies a particularly key place on the scale of priorities of the Saudi community. The present research paper contributes to these debates by investigating the effectiveness of health expenditures in modulating the incidences of CO_2_ emissions on the population’s health status for Saudi Arabia, particularly disability-adjusted life years and infant mortality.

In addition to the foregoing, the empirical strategy of this leading research is founded on the work of Nelson and Phelps (40) which presents health capital as a crucial element of the absorption and diffusion of technology within an economy thus ensuring a higher growth rate. Implicitly, these macroeconomic models assume that health status is the result of investments in the health sector. There would then exist a production function whose output would be the state of health and for input the resources of the health sector. Following this reasoning, the general form of a health production function can be written:


(1) HS=f(H)

Where HS represents the health status and H is the health inputs. Following Omri et al. (4), this study split HI into three elements, namely environment (Env), social (Soc), and economic (Eco). So, we obtain the following function:


(2) HS=f(Env,Soc,Eco)

Since the principal aim of this work is to examine the links among environment, health expenditures, and health status, the economic and social variables are as control variables in the model. Besides, we propose four indicators to measure environmental degradation, specifically *per capita* CO_2_ emissions (COpc), CO_2_ from liquid fuel consumption (COlfc), CO_2_ emissions from electricity and heat production (COehp), and CO_2_ intensity (COint). For the social indicators, we included education. For the economic indicators, we included GDP growth. Likewise, the augmented health outcome’s function can be presented as such:


(3) $$HS_{t}=\beta_{0}+\beta_{1}Env_{t}+\beta_{2}X_{t}+\varepsilon_{t}$$

where t designates the time during the period 1990–2020, HS represents the two indicators of health status, Env specifies the four proxies of CO2 emissions, and X represents the control variables, including GDP growth and education, $\;\beta_{0}$ represents the constant coefficient of the regression and $\beta_{1},\beta_{2}\;$ refer to the coefficient associated with the four proxies of CO2 emissions and the control variables, respectively.

To explore the effectiveness of health expenditures to modulate the adverse effects of CO2 emissions on health status, Eq. (3) could be written as:


(4) $$HS_{t}=\beta_{0}+\beta_{1}Env_{t}+\beta_{2}Exp_{t}+\beta_{3}Env_{t}*Exp_{t}+\beta_{4}X_{t}+\varepsilon_{t}$$

where Exp specifies the three proxies of health expenditure (global, private, and public private). Env*Exp indicates the interactions between the indicators of CO2 emissions and the indicators of health expenditures. $\;\beta_{0}$ represents the constant coefficient of the specification and $\beta_{1},\beta_{2},\beta_{3},\beta_{4}\;$ refer to the coefficient associated with the four proxies of CO2 emissions, the three proxies of health expenditure (global, private, and public private), the interactions between the indicators of CO2 emissions and the indicators of health expenditures and the control variables, respectively. The effect of the interaction between health expenditure and CO_2_ emissions on health status has not been studied extensively as we have done so far. The investigation of this type of interaction will generate elements of the answers for us if health expenditure plays a vital role as a moderator in improving the health status affected by CO2 emissions. In this research paper, we use interactions between the three proxies of health expenditure and the four indicators of CO_2_ emissions that makes us possible to decide whether there is a complementarity between them in improving health status. We expect that CO2 emissions deteriorate the health status [e.g., (4, 24, 27, 28)], whereas health expenditures improve it [e.g., (13, 16, 35, 37)]. For the interactions between health expenditure and CO2 emissions, we expect that health expenditures modulate the negative effects of CO2 emissions on disability-adjusted life years and infant mortality.

### 3.2. Estimation procedures

The estimation procedures of our model start by examining the order of integration of series. This step is essential because the employ of non-stationary variables in regression can generate inefficient coefficients, non-optimal predictions, and unacceptable significance tests. Besides, we speak of a time series as stationary when its mean and variance do not vary with time, otherwise, it is said to be non-stationary. In this study, we apply three stationarity tests: Augmented Dickey and Fuller (41), Phillips and Perron (42), and Kwiatkowski et al. (43) tests. Asymptotic distributions of unit root test statistics were constructed assuming the residual term is white noise. Unlike the simple Dickey & Fuller test, the ADF and PP tests consider the possibility of residual autocorrelation in their construction. The first proposes to control autocorrelation directly in the model by including one or more differentiated autoregressive terms. The second, the PP test, proposed a correction of the OLS estimators and associated Student statistics. This is what motivated the choice of these tests.

When deciding the order of integration, the second step consists of identifying the occurrence of cointegrating relationships. The cointegration tests allow noticing that integrated variables of the same order have the same stochastic tendency and therefore a cointegrating relationship. The notion of cointegration can be defined as a long-term systematic co-movement among two or more variables. Granger’s and Johansen’s tests are well suited for time series. Engle and Granger (44) revealed that it is probable for a linear combination of integrated series of the same order to be stationary with an order of integration strictly lower than that of the variables. The Granger test is based on two steps, the statistical regression between the integrated variables of the same order and the verification of the stationarity of the residuals. Johansen’s method consists in imposing restrictions by cointegration and testing them. To test for cointegration and decide the number of cointegrating relationships, the Johansen (45) test has been widely used. It is this test that we apply here as well.

To determine the long-term coefficients, two methods have been proposed (third step). It is known that the Ordinary Least Squares (OLS) technique does not constantly provide stable estimators. In this context, Kao and Chiang (46) suggested the DOLS technique. This last one does not attach immense value to the heterogeneity of individuals. To solve this problem, Pedroni (47) proposes the FMOLS estimator which makes it possible to consider the heterogeneity of individuals, but also the obstacle of endogeneity and autocorrelation. FMOLS estimator not only delivers efficient estimates in small sample sizes but also makes it possible to deal with the endogeneity problem of the regressors and the problem of autocorrelation. FMOLS estimator not only deliver efficient estimates in small sample sizes, but it also makes it possible to deal with the endogeneity problem of the regressors and the problem of autocorrelation. In other words, the FMOLS estimators were developed with the goal of mitigating the effects of endogeneity bias and serial correlation, which would then make it possible to make typical normal inferences. This is accomplished by the FMOLS estimator through the utilization of a non-parametric adjustment.

### 3.3. Data

**Figure 1:**
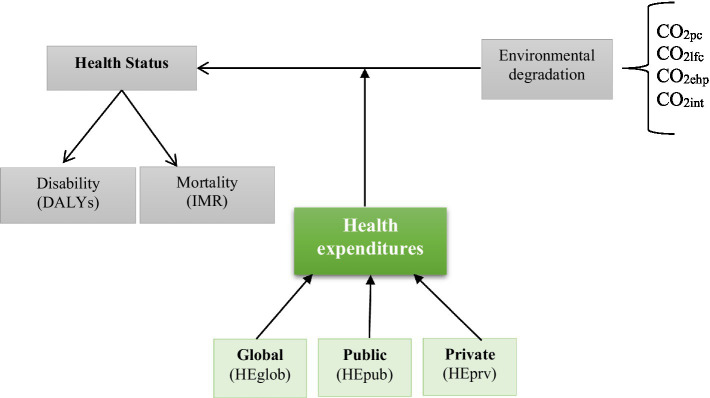
The conceptual model of study.

The present study uses dataset for Saudi Arabia, spanning from 1990 to 2020, to investigate how health expenditures (global, public, and private) could moderate the impact of CO_2_ emissions on health status, particularly infant mortality (IMR) and disability-adjusted life years (DALYs). The period of study was selected given the availability of data for the two main indicators: disability and mortality. We take the logarithm of all used variables. This transformation into logarithm facilitates, on the one hand, the interpretation of the estimated coefficients which are read as elasticities and can control the problem of heteroscedasticity, on the other hand. Indeed, logarithmic transformation makes it possible to resolve or reduce the discrepancies between the variables related to the differences in their units of measurement. Below are the definitions and sources of the variables used (Figure 1).

#### 3.3.1. Dependent variable

The health status’ dependent variable is measured by two indicators, namely DALYs and IMR. DALYs, proposed by the World Bank and the World Health Organization in 1993, measures the overall disease burden. One DALY represents “*a one lost year of healthy life and extends the concept of potential years of life lost due to premature death to include equivalent years of healthy life lost by virtue of being in states of poor health or disability*” (WHO). DALYs data were collected from the Global Burden of Disease Study (GBD). IMR is measured by the rates of infant mortality in 1,000 live births. The dataset on IMR is collected from the World Development Indicators (WDI).

#### 3.3.2. Independent variables

As already mentioned, the principal explanatory variables are as follows.

##### 3.3.2.1. Environmental degradation

CO_2_ emissions are used as a measure of environmental degradation. Four indicators of CO_2_ emissions are considered, namely COpc (metric tons), COint (kg per kg of oil equivalent energy use), COehp (percentage of total fuel combustion), COlfc (percentage of total). Numerous investigations, such as Narayan and Narayan (48), Alimi et al. (49), and Omri et al. (4) have examined the environment-health nexus. Therefore, we assume that CO_2_ indicators have positive impacts on the two indicators of health status (DALYs and IMR).

##### 3.3.2.2. Health Expenditures

The effectiveness of public health spending, compared to other health determinants, becoming essential for decision-makers in charge of developing health policies (4). Previous empirical are inconclusive regarding the influence of health expenditure on health status compared to other factors. They revealed that health expenditure often has a positive impact but is mixed in terms of statistical significance (4, 18, 50, 51). The magnitude of the impact of health expenditures depends on the estimation methods and the control variables included in the models. Moreover, the health policy implications of these studies are not easily transposable to the country level, as there are notable socio-economic, demographic, and epidemiological, as well as political differences between countries, especially between developed and developed countries. In this study, we used three measures of health expenditures, namely global, public, and private health expenditures. We expect that global, public, and private health expenditures improve the population’s health status in Saudi Arabia.

#### 3.3.3. Control variables

Economic growth and education are included in the model as control variables.

##### 3.3.3.1. Education

Investment in human capital in the form of better health status and higher levels of education is the most effective way to bring about higher productivity (52–55). Education may determine many decisions that influence the quality of life, including choosing a job, being able to choose a healthy diet and avoiding unhealthy habits, using medical care effectively, and so on. This research includes this variable to looking at how education affects health outcomes in Saudi Arabia. We expect that education (Educ) in Saudi Arabia contributes to the improvements of health outcomes.

##### 3.3.3.2. GDP growth

Recent years have been marked by abundant literature on the linkage between many measures of growth and a variety of health indicators (4, 56–58). This study uses GDP growth in annual percentage as a measure of economic growth. We expect that increasing economic growth (EG) improves health status in Saudi Arabia.

## 4. Results and discussion

**Figure 2:**
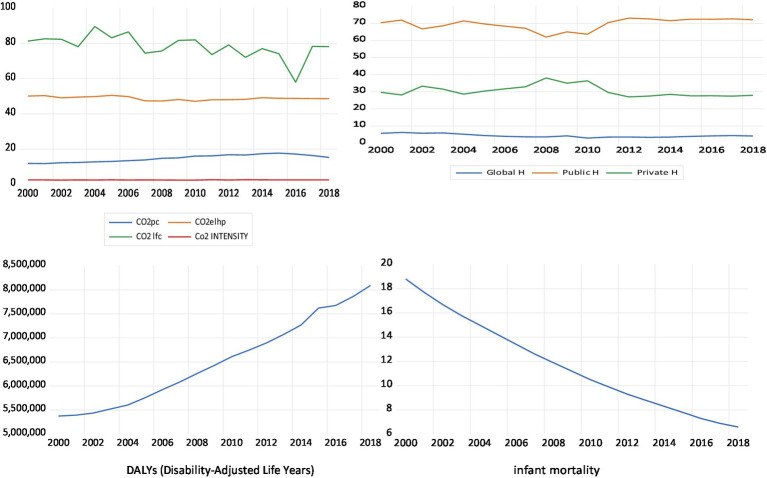
Plots of the main variables.

The descriptive statistics of the used are presented in Table 1 and Figure 2, which show that, during the study’s time period, the value of infant mortality rates range from around 6.6% to around 18.8% per 1,000 live births, whereas the number of healthy years lost ranges from around 5.3 million years to around 8 million years. Bindawas and Vennu (59) argue, in this context, that out of more than 20 million Saudi citizens surveyed, 667,280 of them reported being disabled, representing a prevalence rate of 3,326 out of 100,000 citizens. The values of the indicators of CO_2_ emissions range from around 11.7, 47, 58, and 2.3 to around 17.7, 50.4, 89.5, and 2.6 for COpc, COehp, COlfc, and COint, respectively. The values of the two main indicators of health expenditures range from around 62% and 27% to around 73% and 37% of current health expenditure for, respectively, public and private health expenditures. This table also reports a strong correlation between DALYs and IMR (0.928) and between the indicators of CO_2_ emissions of up to 0.89. These highest correlations allow us to include them separately in the estimated models to avoid the problem of multicollinearity. The correlations between the two indicators of health status and the indicators of CO_2_ emissions are positive, whereas their correlations with the indicators of health expenditures are negative.

**Table 1:** Descriptive statistics and pairwise correlations.

	IMR	DALYs	COpc	COehp	COlfc	COint	HEglob	HEpub	HEprv	EG	Educ
Mean	11.720	6506111.1	14.748	48.810	78.269	2.469	4.382	69.515	30.485	3.880	40.961
Standard deviation	3.825	902508.7	2.039	1.153	7.086	0.063	0.971	3.300	3.300	5.431	16.130
Min	6.600	5374414.4	11.724	47.139	57.924	2.367	2.971	61.932	27.018	−3.763	22.321
Max	18.800	8088708.5	17.691	50.486	89.531	2.598	6.262	72.932	37.068	15.193	69.698
IMR	1										
DALYs	−0.928	1									
COpc	0.769	0.729	1								
COehp	0.743	0.683	0.824	1							
COlfc	0.679	0.702	0.856	0.798	1						
COint	0.207	0.251	0.803	0.812	0.891	1					
HEglob	−0.755	−0.798	−0.752	−0.761	−0.571	−0.067	1				
HEpub	−0.588	−0.396	−0.369	−0.399	−0.529	−0.545	0.080	1			
HEprv	−0.588	−0.396	−0.369	0.399	−0.529	−0.545	0.080	0.999	1		
EG	−0.691	−0.740	0.736	0.658	0.617	−0.377	0.449	0.283	0.283	1	
Educ	−0.791	−0.757	−0.318	−0.188	−0.218	−0.157	0.736	0.012	0.614	0.265	1

We started our empirical analysis by checking the stationarity of the variables used. Accordingly, different unit root tests are employed, namely ADF (1981), PP (1988), and KPSS (1992). Table 2 reported the results of these tests and showed that all variables are integrated into the first difference, indicating that our variables are integrated in order one (I (1)). Hence, it is possible to check the presence of long-run relationships between the different variables using Johansen’s (45) cointegration test. Table 3 reported the results of this test and showed that, at rank = 0, the statistics of the trace test are greater than the critical value, showing the existence of long-run relationships among variables, i.e., the used variables are cointegrated. The next step consists of estimating the long-run relationships using the FMOLS estimator.

**Table 2:** Results of unit root tests.

Variables	ADF		PP		KPSS	
	Level	1st difference	Level	1st difference	Level	1st difference
IMR	0.711	−5.263^*^	−1.7992**	−1.634^***^	0.128^*^	0.152^**^
DALYs	−4.119^**^	−3.938^**^	−8.470*	−3.954^**^	0.142^*^	0.138^*^
COpc	1.603	−8.520^*^	1.464	−1.892^***^	0.150^*^	0.170^*^
COehp	−1.556	−3.487^***^	−1.662	−3.884^**^	0.113^*^	0.242^*^
COlfc	−2.789	−4.884^*^	−2.300	−7.178^*^	0.135^**^	0.167^*^
COint	−4.022^**^	−8.015^*^	−4.031**	−15.176^*^	0.123^*^	0.091^*^
HEglob	−1.385	−3.853^**^	−1.385	−10.513^*^	0.177^**^	0.122^**^
HEpub	−2.100	−4.619^*^	−2.081	−4.619^*^	0.128^*^	0.068^*^
HEprv	−2.100	−4.619^*^	−2.081	−4.619^*^	0.128^*^	0.068^*^
Economic growth	−1.541	−4.109^**^	−1.541	−4.114^**^	0.124^***^	0.121^**^
Education	−0.286	−6.346^*^	−0.076	−6.346^*^	0.240^**^	0.122^**^

*, **, and *** indicate the significance level at 1%, 5%, and 10%, respectively.

**Table 3:** Johansen’s cointegration test.

	Models	Infant mortality rate (IMR)						Disability-adjusted life year (DALYs)					
		Global (HEglob)Model 1		Public (HEpub)Model 2		Private (HEprv)Model 3		Global (HEglob)Model 1		Public (HEpub)Model 2		Private (HEprv)Model 3	
	Rank = 0^*^	Trace test	Critical value (5%)	Trace test	Critical value (5%)	Trace test	Critical value (5%)	Trace test	Critical value (5%)	Trace test	Critical value (5%)	Trace test	Critical value (5%)
COpc	0	203.300	69.818	172.437	69.818	168.679	69.818	164.023	69.818	161.616	69.818	161.197	69.818
COehp	0	173.560	69.818	143.602	69.818	143.437	69.818	140.931	69.818	153.238	69.818	152.002	69.818
COlfc	0	165.185	69.818	148.168	69.818	149.088	69.818	148.830	69.818	169.296	69.818	167.894	69.818
COint	0	157.052	69.818	150.196	69.818	148.720	69.818	143.987	69.818	150.991	69.818	147.383	69.818

^*^ indicates the rejection of the null hypothesis at 5% significance level.

Tables 4, 5 present the long-run estimates for IMR and DALYs. It is clear from Table 4 that infant mortality elasticities of the indicators of CO2 emissions are positive and significant in all the estimated models, ranging from 0.098% to 0.215% for the models of the global health expenditure, from 0.122% to 0.177% for the models of the public health expenditures, and from 0.116% to 0.203% for the models of the private health expenditures, meaning that carbon emissions increase infant mortality in Saudi Arabia. This result is in line with Omri et al. (4) who examined the effects of environmental quality and R&D on healthcare status in the same country and found that environmental degradation increases mortality and decreases life expectancy. To moderate the negative effect of environmental degradation on the population’s health status, the authors called the Saudi policymakers to support the efforts of R&D and to review and increase their spending on health. In the same direction, Mutizwa and Makochekanwa (24) investigated the influence of environmental degradation (CO2) on IMR for 12 SADC (Southern African Development Community) countries and found that environmental indicators contribute to 38% of infant mortality. However, Chuang et al. (25) explored the interlink between ecological footprints, environmental degradation, IMR, and under-five mortality rate and found that ecological footprints and environmental degradation did not influence the link between economic features and health status. This table also shows that, as expected, health expenditures are negatively correlated with the rates of infant mortality, ranging from −0.302% to −0.412% for the models of the global health expenditure, from −0.492% to −0.602% for the models of the public health expenditure, and from −0.094% to −0.122% for the models of the private health expenditure. The positive contributions of health expenditures on improving the health status confirm the findings of Bokhari et al. (60) who investigated the contribution of health spending and income on health status and found that these variables are necessary factors for improving the population’s health status. They argued that an increase in health expenditures would not indeed lead to enhancing the population’s health status except these increases were accompanied by institutions, tools, and policies that correctly allocate funds appropriately and characterize intra and intersectoral needs. In the same direction, Similarly, Rahman and Alam (50) also found that private and public health expenditures decrease infant mortality and only private health expenditures reduce the crude death rates. They explain the insignificant impact of public health spending on the crude death rate by the inefficient use of these funds due to corruption and ineffective governance. From these statistics, it is clear that the contribution of public health expenditures (−0.492 to −0.602) is higher than private health expenditures (−0.094 to −0.122), indicating that, in Saudi Arabia, public health expenditures are more efficient than private health expenditure in reducing infant mortality. Therefore, the government may provide enough necessary financial resources to improve the population’s health status. This result contradicts the results of Raeesi et al. (18) who explored the link among PPHE and three health outcomes indicators (IMR, U5MR, and life expectancy) for 25 nations and found that the influence of private health expenditure on the health outcomes indicators was bigger than public health expenditure. Moreover, we emphasize one of the gaps existing in the previous research, i.e., examining the efficiency of global, public, and private health expenditures on modulating the negative effects of carbon emissions on infant mortality. Table 4 also shows that the effects of the interactions between health expenditures and carbon emissions on infant mortality are negative only for the specifications related to public health expenditure, ranging from −0.129% to −0.144%. This result indicates that increasing public health expenditures is effective to moderate the negative effects of CO2 emissions on infant mortality. In other words, this research paper concludes that public health expenditures play a significant role as a moderator in enhancing the health status affected by CO2 emissions. This result confirms the findings of Ganda (15), Bu and Ali (22), and Li et al. (32), who found that public health expenditures are effective in reducing emissions, which, in turn, reduces infant mortality (4). Therefore, policymakers in Saudi Arabia should increase the government spending on health to moderate the influence of carbon emissions on the population’s health status, particularly infant mortality. Finally, economic growth and education are found to have positive impacts on reducing infant mortality.

**Table 4:** Results of the impacts of health expenditure and environmental degradation on infant mortality.

Independent variables	Dependent variable: Infant mortality (IMR)											
	Global (HEglob)Model 1				Public (HEpub)Model 2				Private (HEprv)Model 2			
	1	2	3	4	5	6	7	8	9	10	11	12
COpc	0.215^**^(0.011)	-	-	-	0.177^*^(0.003)	-	-	-	0.203^*^(0.000)	-	-	-
COehp	-	0.188^*^(0.000)	-	-	-	0.169^*^(0.000)	-	-	-	0.171^**^(0.019)	-	-
COlfc	-	-	0.098^***^(0.051)	-	-	-	0.126^**^(0.038)	-	-	-	0.116^***^(0.066)	-
COint	-	-	-	0.135^**^(0.020)	-	-	-	0.122^**^(0.018)	-	-	-	0.157^**^(0.040)
HEglob	−0.383^*^(0.000)	−0.329^*^(0.000)	−0.412^*^(0.000)	−0.302^*^(0.000)	-	-	-	-	-	-	-	-
HEpub	-	-	-	-	−0.535^*^(0.000)	−0.590^*^(0.000)	−0.602^*^(0.000)	−0.492^*^(0.000)	-	-	-	-
HEprv	-	-	-	-	-	-	-	-	−0.122^*^(0.000)	−0.109^**^(0.014)	−0.097^**^(0.012)	−0.094^**^(0.036)
COpc * HEglob	−0.061(0.014)	-	-	-	-	-	-	-	-	-	-	-
COehp * HEglob	-	−0.077(0.138)	-	-	-	-	-	-	-	-	-	-
COlfc * HEglob	-	-	−0.058(0.222)	-	-	-	-	-	-	-	-	-
COint * HEglob	-	-	-	−0.087(0.113)	-	-	-	-	-	-	-	-
COpc * HEpub	-	-	-	-	−0.139^*^(0.002)	-	-	-	-	-	-	-
COehp * HEpub	-	-	-	-	-	−0.154^*^(0.000)	-	-	-	-	-	-
COlfc * HEpub	-	-	-	-	-	-	−0.129^**^(0.018)	-	-	-	-	-
COint * HEpub	-	-	-	-	-	-	-	−0.144^*^(0.000)	-	-	-	-
COpc * HEprv	-	-	-	-	-	-	-	-	−0.086(0.122)	-	-	-
COehp * HEprv	-	-	-	-	-	-	-	-	-	−0.043(0.237)	-	-
COlfc * HEprv	-	-	-	-	-	-	-	-	-	-	−0.011(0.428)	-
COint * HEprv	-	-	-	-	-	-	-	-	-	-	-	−0.082(0.110)
Economic growth	−0.425^*^(0.000)	−0.289^*^(0.000)	−0.322^*^(0.000)	−0.299^*^(0.000)	−0.440^*^(0.000)	−0.318^*^(0.000)	−0.400^*^(0.000)	−0.302^*^(0.000)	−0.391^*^(0.000)	−0.274^*^(0.000)	−0.336^*^ (0.000)	−0.279^*^(0.000)
Education	−0.182^*^(0.000)	−0.152^**^(0.034)	−0.201^*^(0.000)	−0.114^***^(0.052)	−0.177^*^(0.000)	−0.139(0.112)	−0.198^*^(0.000)	−0.123(0.133)	−0.211^*^(0.000)	−0.209^*^(0.000)	−0.166^**^(0.028)	−0.184^*^(0.000)
Constant	9.012^*^(0.000)	10.328^*^(0.000)	6.870^*^(0.000)	3.318^*^(0.000)	9.523(0.000)	13.244^*^(0.000)	10.692^*^(0.000)	8.440^*^(0.000)	7.254^*^(0.000)	8.241^*^(0.000)	9.288^*^(0.000)	6.052^*^(0.000)

*, **, and *** indicate the significance levels at 1%, 5%, and 10%, respectively.

**Table 5:** Results of the impacts of health expenditure and environmental degradation on disability.

Independent variables	Disability-adjusted life year (DALYs)											
	Global (HEglob) Model 1				Public (HEpub) Model 2				Private (HEprv) Model 2			
	1	2	3	4	5	6	7	8	9	10	11	12
COpc	0.179^*^(0.001)	-	-	-	0.162^*^(0.000)	-	-	-	0.192^*^(0.000)	-	-	-
COehp	-	0.197^*^(0.000)	-	-	-	0.184^*^(0.000)	-	-	-	0.214^*^(0.000)	-	-
COlfc	-	-	0.083^**^(0.030)	-	-	-	0.109^**^(0.027)	-	-	-	0.099^***^(0.053)	-
COint	-	-	-	0.154^*^(0.009)	-	-	-	0.183^*^(0.004)	-	-	-	0.129^**^(0.019)
HEglob	−0.106(0.110)	−0.049(0.241)	−0.088(0.125)	−0.105(0.147)	-	-	-	-	-	-	-	-
HEpub	-	-	-	-	−0.226^*^(0.000)	−0.296^*^(0.000)	−0.368^*^(0.000)	−0.329^*^(0.000)	-	-	-	-
HEprv	-	-	-	-	-	-	-	-	−0.113(0.100)	−0.093(0.142)	−0.101(0.114)	−0.084(0.168)
COpc * HEglob	−0.056(0.218)	-	-	-	-	-	-	-	-	-	-	-
COehp * HEglob	-	−0.122(0.107)	-	-	-	-	-	-	-	-	-	-
COlfc * HEglob	-	-	−0.086(0.149)	-	-	-	-	-	-	-	-	-
COint * HEglob	-	-	-	−0.077(0.187)	-	-	-	-	-	-	-	-
COpc * HEpub	-	-	-	-	−0.188^*^(0.000)	-	-	-	-	-	-	-
COehp * HEpub	-	-	-	-	-	−0.172^**^(0.015)	-	-	-	-	-	-
COlfc * HEpub	-	-	-	-	-	-	−0.143^**^(0.010)	-	-	-	-	-
COint * HEpub	-	-	-	-	-	-	-	−0.118^**^(0.024)	-	-	-	-
COpc * HEprv	-	-	-	-	-	-	-	-	−0.049(0.298)	-	-	-
COehp * HEprv	-	-	-	-	-	-	-	-	-	−0.059(0.325)	-	-
COlfc * HEprv	-	-	-	-	-	-	-	-	-	-	−0.108(0.112)	-
COint * HEprv	-	-	-	-	-	-	-	-	-	-	-	−0.097(0.142)
GE	−0.425^*^(0.000)	−0.289^*^(0.000)	−0.322^*^(0.000)	−0.299^*^(0.000)	−0.440^*^(0.000)	−0.318^*^(0.000)	−0.400^*^(0.000)	−0.302^*^(0.000)	−0.391^*^(0.000)	−0.274^*^(0.000)	−0.336^*^(0.000)	−0.279^*^(0.000)
Educ	−0.182^*^(0.000)	−0.152^**^(0.034)	−0.201^*^(0.000)	−0.114^***^(0.052)	−0.177^*^(0.000)	−0.139(0.112)	−0.198^*^(0.000)	−0.123(0.133)	−0.211^*^(0.000)	−0.209^*^(0.000)	−0.166^**^(0.028)	−0.184^*^(0.000)
Constant	9.012^*^(0.000)	10.328^*^(0.000)	6.870^*^(0.000)	3.318^*^(0.000)	9.523(0.000)	13.244^*^(0.000)	10.692^*^(0.000)	8.440^*^(0.000)	7.254^*^(0.000)	8.241^*^(0.000)	9.288^*^(0.000)	6.052^*^(0.000)

*, **, and *** indicate the significance levels at 1%, 5%, and 10%, respectively.

As mentioned above, Table 5 reported the results of the empirical associations among health expenditures, CO_2_ emissions, and disability-adjusted life years, showing that DALYs elasticities of the indicators of CO_2_ emissions are positive and significant, ranging from 0.083% to 0.197% for the models of the global health expenditure, from 0.109% to 0.184% for the models of the public health expenditures, and from 0.099% to 0.214% for the models of the private health expenditures, meaning that carbon emissions increase the number of healthy years lost in Saudi Arabia, confirming the findings of Owusu and Sarkodie (12) who investigated the contributions of air pollution on DALYs, Mortality, and welfare for 195 countries and they found significant positive and negative impacts of air pollution on health status (DALYs, mortality, and premature deaths) and economic development, respectively. They documented that many high-income countries have recently made efforts to mitigate environmental pollution, which declines DALYs, mortality, and welfare cost. It is reported that environmental degradation, induced by air pollution, contributes to about 103 million DALYs and 4 million global deaths in the year 2015 (61). Table 5 also shows that, as expected, health expenditures are negatively correlated with DALY only for the specifications related to public health expenditure, ranging from −0.226% to −0.368% for the models of the public health expenditures. This result means that the number of years lost due to disability is negatively correlated with the increase of health expenditures, particularly public health expenditures, confirming the findings of Danovi et al. (39) who examined the role of health expenditures in achieving a healthcare sustainability for the United States, the BRICS countries, and the European Union. Their findings revealed a positive association between the number of years lost due to disability and health expenditures. They argued that more resources should be invested not only to diminish the years lost due to disability but also to lower severity, occurrence, and duration of diseases that cause morbidity, comorbidity and polimorbidity, but not mortality. In addition, we focus on another shortcoming in prior studies, i.e., testing the effectiveness of global, public, and private health expenditures on modulating the negative impacts of carbon emissions on the number of years’ loss of healthy life (DALYs). Table 5 also reported that the effects of the interaction between health expenditure and carbon emissions on DALYs are negative and statistically significant only for the specifications related to public health expenditures, ranging from −0.118% to −0.188%. This result means that increasing public health expenditures moderate the negative influence of carbon emissions on infant mortality, confirming the findings of Ganda (15), Bu and Ali (22), and Li et al. (32), who found that public health expenditures are effective in reducing emissions, which, in turn, decreases DALYs (12, 61). Therefore, policymakers in Saudi Arabia should increase the government spending on health to moderate the effects of carbon emissions on the number of years’ loss of healthy life (DALYs). Finally, most of the control variables do not have the expected signs. Economic growth and education do not have significant impacts on reducing DALYs.

## 5. Conclusion and policy implications

Environmental degradation and climate change and their connections to population health status have recently received much attention. The historical trends of premature deaths, infant mortality, and DALYs costs have a lingering influence on the future development of countries. Accordingly, this study uses dataset for Saudi Arabia to investigate the effectiveness of health expenditures to modulate the negative impacts of CO_2_ emissions on health status, particularly disability-adjusted life years and infant mortality. Four indicators of CO_2_ emissions (COpc, COehp, COlfc, and COint) and three categories of health expenditures (HEglob, HEpub, and HEprv) are included in the analysis. Necessary econometric procedures, including unit root test, Johansen’s cointegration test, and FMOLS method, are used. The empirical results show that (i) unconditional positive effects of CO2 emissions on increasing DALYs and infant mortality; (ii) conditional negative impacts of public health expenditures on DALYs and infant mortality, whereas global and private health expenditure contributes only on reducing infant mortality; (iii) public health expenditures are more effective than private health expenditures to reduce infant mortality; (iv) the effects of the interactions between the indicators of both health expenditures and CO2 emissions on DALYs and infant mortality are negative and significant only for the specifications related to public health expenditure, indicating that this later could be employed as a policy or conditional variable that modulates the adverse effects of environmental degradation on the population’s health status. In fact, it has been found that environmental indicators contribute to increase in infant mortality due to environmental degradation. Further, the output of the research paper indicates that increasing public health expenditures is effective to moderate the negative effects of CO2 emissions on infant mortality. In other words, our conclusion is that public health expenditures play a significant role as a moderator in enhancing the health status affected by CO2 emissions. Therefore, policymakers in Saudi Arabia should increase the government spending on health to moderate the influence of carbon emissions on the population’s health status, particularly infant mortality.

In light of the above findings, some policy recommendations have been suggested. First, since our findings revealed that public health expenditure could modulate the incidences of environmental degradation on health status, particularly, infant mortality, decision makers in Saudi Arabia are called to review and augment their health expenditures to effectively curbing CO_2_ emissions, hence, promoting a healthy environment. Second, CO_2_ emissions are found to have negative effects on population health status by generating various bacteria and viruses, which are the causes of different diseases, such as heart problems, bronchitis, and flu-related illnesses like coronavirus (COVID-19) and many other life-threatening conditions. Therefore, urgent measures and appropriate policies toward a low-carbon environment should be taken to foster longevity. Finally, Economic progress allows the population to profit from more developed healthcare facilities; however, the negative externalities of economic activities, particularly environmental pollution, can harm public health. Accordingly, smart, sustainable, and health-friendly economic growth policy reforms will be beneficial in increasing the lifespan of the population. Moreover, it is significant to underline that our work makes interesting contributions to the effectiveness of health expenditures to moderate the adverse effects of carbon emissions on health status, but this does not mask that some other variables like renewable energy, governance, environmental taxation, among others, may be included as moderator variables. Future research could extend this work by considering the roles of the above-mentioned variables.

## Data availability statement

The original contributions presented in the study are included in the article/supplementary material, further inquiries can be directed to the corresponding author.

## Author contributions

AO, BK, and MK: software and writing—original draft preparation. All authors contributed to the article and approved the submitted version.

## Funding

This research was funded by the King Salman Center for Disability Research, grant no KSRG-2022-051.

## Conflict of interest

The authors declare that the research was conducted in the absence of any commercial or financial relationships that could be construed as a potential conflict of interest.

## Publisher’s note

All claims expressed in this article are solely those of the authors and do not necessarily represent those of their affiliated organizations, or those of the publisher, the editors and the reviewers. Any product that may be evaluated in this article, or claim that may be made by its manufacturer, is not guaranteed or endorsed by the publisher.

## Appendix

**Table A.1.** Data source and expected signs of each variable.

**Table:**

**Indicators adopted**	**Variables**	**Expression**	**Sources**
**Health status indicators**	All-cause DALYs (years per 100,000 inhabitants), all ages.	DALYs	GBD (2019)
	Infant mortality as rate per 1,000 live births.	IMR	WDI (2021)
**Environmental degradation indicators**	Carbon dioxide *per capita* (metric tons)	CO_pc_	WDI (2021)
	Carbon dioxide intensity (kg per kg of oil equivalent energy use)	CO_int_	WDI (2021)
	Carbon dioxide emissions from electricity and heat production, total (% of total fuel combustion)	COehp	WDI (2021)
	Carbon dioxide emissions from liquid fuel consumption (% of total).	COlfc	WDI (2021)
**Economic and social indicators**	Current health expenditures (%of GDP)	HEglob	WDI (2021)
	Public Health Expenditures (domestic general government health expenditure (%) of current health expenditure)	HEpub	WDI (2021)
	Private Health Expenditures (Domestic private health expenditure (%) of current health expenditure)	HEprv	WDI (2021)
**Control variables**	FDP growth (%)	EG	WDI (2021)
	Gross enrollment rate in tertiary education (%).	Educ	WDI (2021)
